# Development, implementation, and scalability of the Family Engagement in Research Course: a novel online course for family partners and researchers in neurodevelopmental disability and child health

**DOI:** 10.1186/s40900-024-00615-w

**Published:** 2024-08-05

**Authors:** Andrea Cross, Alice Kelen Soper, Donna Thomson, Connie Putterman, Dayle McCauley, Samantha K. Micsinszki, Rachel Martens, Patricia Solomon, Lorraine Carter, James N. Reynolds, Olaf Kraus de Camargo, Jan Willem Gorter

**Affiliations:** 1 CanChild Centre for Childhood Disability Research, McMaster University, Hamilton, ON Canada; 2 Department of Pediatrics, McMaster University, Hamilton, ON Canada; 3 School of Rehabilitation, McMaster University, Hamilton, ON Canada; 4 Centre for Addiction and Mental Health, Toronto, ON Canada; 5 Azrieli Accelerator Program, University of Calgary, Calgary, AB Canada; 6 McMaster Continuing Education, McMaster University, Hamilton, ON Canada; 7 Department of Biomedical and Molecular Sciences, Queen’s University, Kingston, ON Canada; 8 Department of Rehabilitation, Physical Therapy Science and Sports, UMC Utrecht Brain Center, University Medical Center Utrecht, Utrecht, the Netherlands

**Keywords:** Family engagement in research, Online education, Family-researcher partnerships, Training, Continuing education, Co-design, Child health research, Neurodevelopmental disability, Patient-oriented research, Co-learning, Community building

## Abstract

**Background:**

Since 2011 when the Canadian Institutes of Health Research launched the Strategy for Patient Oriented Research, there has been a growing expectation to embed patient-oriented research (POR) in the health research community in Canada. To meet this expectation and build capacity for POR in the field of neurodevelopmental disability and child health, in 2017 researchers and family leaders at CanChild Centre for Childhood Disability Research, McMaster University partnered with Kids Brain Health Network and McMaster Continuing Education to develop and implement a 10-week online Family Engagement in Research (FER) Course.

**Main text:**

From its inception, the FER Course has been delivered in partnership with family leaders and researchers. The FER Course is innovative in its co-learning and community building approach. The course is designed to bring family partners and researchers together to co-learn and connect, and to develop competency and confidence in both the theory and practice of family engagement in research. Coursework involves four live online group discussions, individual review of course materials, weekly group activities, and a final group project and presentation. Upon completion of the FER Course, graduates earn a McMaster University micro-credential.

**Conclusions:**

To meet a need in building capacity in POR, a novel course in the field of neurodevelopmental disability and child health has been co-created and delivered. Over six years (2018–2023), the FER Course has trained more than 430 researchers and family partners across 20 countries. A unique outcome of the FER Course is that graduates expressed the wish to stay connected and continue to collaborate well beyond the course in turn creating an international FER Community Network that continues to evolve based on need. The FER Course is creating a growing international community of researchers, trainees, self-advocates, and family partners who are championing the implementation of meaningful engagement in neurodevelopmental disability and child health research and beyond. The course is internationally recognized with an established record of building capacity in POR. Its uptake, sustainability, and scalability to date has illustrated that training programs like the FER Course are necessary for building capacity and leadership in family engagement in research.

## Abstract (plain language summary)

In the last two decades there has been a clear commitment in Canada (and the world) to include patients and their families in health research—a process called patient-oriented research or as we refer to it—family engagement in research. In 2011, the Canadian Institutes of Health Research introduced the Strategy for Patient-Oriented Research to make this happen. To support POR in neurodevelopmental disability and child health, CanChild Centre for Childhood Disability Research teamed up with Kids Brain Health Network and McMaster Continuing Education. Together, a team of family caregivers and researchers co-created the Family Engagement in Research (FER) Course, a 10-week online course.

The purpose of the FER Course is for researchers and family partners to learn about family engagement principles and how to use them in research. The course covers core areas in family engagement including how to find each other, how families and researchers can work together, and ways to overcome common challenges in research partnerships. The course uses online group sessions, discussion boards, and various resources such as research papers and videos. Through a group project, family partners and researchers collaborate to create a resource on family engagement. Completing the FER Course researchers and family members earn a McMaster University micro-credential and become part of a growing global community of FER Course graduates.

Over six years (2018–2023), the FER Course has trained more than 430 researchers and family partners from 20 countries. The course has strengthened capacity in family engagement and is building a worldwide community of researchers, trainees, self-advocates, and family partners who are dedicated to improving neurodevelopmental disability and child health research through meaningful engagement.

## Background

Meaningful engagement of patients as equal partners in health research is essential to achieve relevant and meaningful research outcomes [1–4]. ‘Patients’ in this context are defined as “an overarching term inclusive of individuals with personal experience of a health issue and informal caregivers, including family and friends” [5]. The Canadian Institutes of Health Research (CIHR) defines the engagement of patients in research, or Patient Oriented Research (POR), as “a continuum of research that engages patients as partners, focusses on patient-identified priorities, and improves patient outcomes” [5].

Since the 2011 launch of CIHR’s Strategy for Patient Oriented Research (SPOR), there has been a growing expectation to implement a POR approach; however, this expectation has not been matched with sufficient training, education, mentorship, and implementation support. Over the last decade, various frameworks, resources, and websites have been developed to increase knowledge and awareness of POR [6–8]. However, there is a paucity of training programs available to researchers and patients in which they co-learn and develop working relationships [9, 10]. As of January 2024, there are only a few online synchronous training programs that exist in Canada (e.g., Ontario SPOR Support Unit Master Class on Patient Oriented Research [11], Knowledge Translation Program Partners in Research (PiR) Program [12], Alberta SPOR Foundations of Patient Oriented Research Certificate) [13]. All these programs are general and broadly applicable to patients and researchers seeking partnership in all areas of health research. None of these programs include an experiential component where patients and researchers get to apply their learning and work together on a project.

In the field of neurodevelopmental disability (NDD) and child health research, family engagement in research (FER) falls within the area of POR and focuses broadly on the collaboration and partnership with family partners (parents, caregivers, grandparents, siblings, close family friends, etc.) throughout the entire research process [14]. In 2017, our team of family partners and health service researchers recognized that, without a community of trained family partners and researchers dedicated to advancing FER, the goal of meaningful engagement of people with lived experience as equal partners in NDD and child health research would be unrealizable. As defined by Hamilton and colleagues, the guiding principles of meaningful engagement are* “*involving patients early (contributing to research questions and design), inclusiveness, co-learning, co-building of knowledge and providing support” (p. 397) [15].

Given limited training opportunities and a lack of experiential opportunities, our team of family partners and researchers saw a need to develop a course for both families and researchers within the field of NDD and child health. As an integrated team our objective was to create a community of family partners and researchers who would learn both the theory and practice of FER in equal partnership through a co-learning and community building approach. In this co-learning approach, researchers and family partners learn from each other and, through sharing their perspectives and experiences, build trust and mutual respect [16]. In 2018, our vision was realized when we launched the Family Engagement in Research (FER) Course at McMaster University [17].

## The Evolution of the FER Course

The purpose of this paper is to describe the conception, development, implementation, sustainability, and scalability of the FER Course through its first six years (September 2017 – December 2023).

### Course conception

The FER Course grew out of a long-standing partnership between CanChild Centre for Childhood Disability Research [18] and Kids Brain Health Network (KBHN) [19]. Both CanChild and KBHN have a common focus on generating knowledge through research and translating it into practice to improve the lives of children with NDD and their families. The idea of creating the FER Course originated with three researchers at CanChild and two parent partners during an informal meeting at a KBHN Conference in October 2017. With a mutual interest in building capacity in FER, we identified a gap in training and education for researchers and family partners to develop competency and confidence in both the theory and practice of FER. We then formed a team along with a senior researcher and leader in the field of educational innovation and problem-based learning at McMaster University, submitted a project grant application, and in 2018 were awarded funding from KBHN to develop and pilot the FER Course with two cohorts of 16 learners (8 family partners and 8 researchers).

### Course development

Our goal was to launch the first FER Course (September 2018) which gave us eight months to develop the course. From the outset, it was important to us that the FER Course met the training needs of families of children with NDD and researchers in the field of NDD. In February 2018, we hosted an initial Planning Meeting at McMaster University. The purpose of this half-day meeting was to introduce the idea of the FER Course to family partners, trainees, and researchers, and to gather feedback on the course content and design. Twenty-one people (including five trainees, six researchers, seven families, and three members of the KBHN Research and Training Committee) gathered for discussion and collaboration. Feedback was used to inform the overall course goals and learning objectives.

Following the Planning Meeting, three members of our team collaborated to co-create the course content over six months. Employed as paid consultants at this stage, two family partners divided lead writer roles for each week of the course content equally with a post-doctoral fellow research trainee. Informed by members of our team’s own lived experience in research partnership and the POR literature, the team co-wrote the weekly content overview and learning objectives, and then curated readings and resources to support meeting these objectives. Weekly content was reviewed and revised by multiple team members in a collaborative manner. From the beginning, it was important to our team that the FER Course was certified by McMaster University. Through a partnership with the Director of McMaster Continuing Education, the FER Course was submitted to the university’s governance committees and was approved as a Certificate of Completion.

#### Course format

The FER Course is a 10-week (~ 30 h) online course that is designed to bring family partners and researchers together to co-learn, connect, and to build trusting relationships in a supportive learning environment. The course is co-facilitated by family leaders and researchers and welcomes learners from diverse backgrounds and all levels of FER experience from across Canada and internationally. Coursework involves four online synchronous (in real-time) group discussions using Zoom video conferencing platform [20], individual review of weekly course materials, participation in weekly group activities (e.g., answering questions and posting peer responses on a discussion board), and completion of a group project and presentation. All course materials are stored on an online learning platform [21], to allow for easy access and participation of learners from various geographic locations. The course format reflects the intention to maintain families’ identities (i.e., there is no intention to turn them into researchers) but rather to bridge cultures and divides between lived experience and research.

#### Course content

The FER Course covers key principles and frameworks of FER alongside discussions of the practicalities and challenges of research partnership. Weekly topics include the importance of FER, how to find each other, how to work in partnership throughout the research process, barriers and facilitators to engagement, ethics surrounding engagement, and tools and resources to support and evaluate engagement activities (see Table 1 for weekly course schedule). Weekly course content includes peer-reviewed manuscripts, online blogs and websites, podcasts, and videos. We have also integrated two simulation videos developed by colleagues at McMaster University and Holland Bloorview Kids Rehabilitation Hospital. These co-designed videos [22] aim to increase learners’ knowledge and skill of meaningful engagement in research by watching and discussing challenging situations that can arise when partnering in research. Informed by universal design for learning principles [23] and consultation with an accessibility expert, multiple formats of educational materials are incorporated into the FER Course to support a variety of learning styles (e.g., podcasts, videos, readings). Each year course materials are reviewed and updated to respond to learner feedback and to reflect the growing body of research and resources in POR. Overtime key additions to the course content included adding a selection of FER tools and resources created by FER Course learners for their group project, and plain language summaries of the required weekly readings.

**Table 1:** Weekly FER Course schedule

Week 1	Family Engagement in Research: What do we really mean?
Week 2	Family Engagement in Research: Why is it important?
Week 3	Building an integrated research team: How can we find each other?
Week 4	Building an integrated research team: How can we work together?
Week 5	Roles and responsibilities of families and researchers
Week 6	Ethics of family engagement in research
Week 7	Barriers and facilitators to family engagement
Week 8	Family engagement tools and resources
Week 9	Evaluation of family engagement activities
Week 10	Next steps: Building a community for family-researcher partnerships

#### Course assessments

**Fig. 1:**
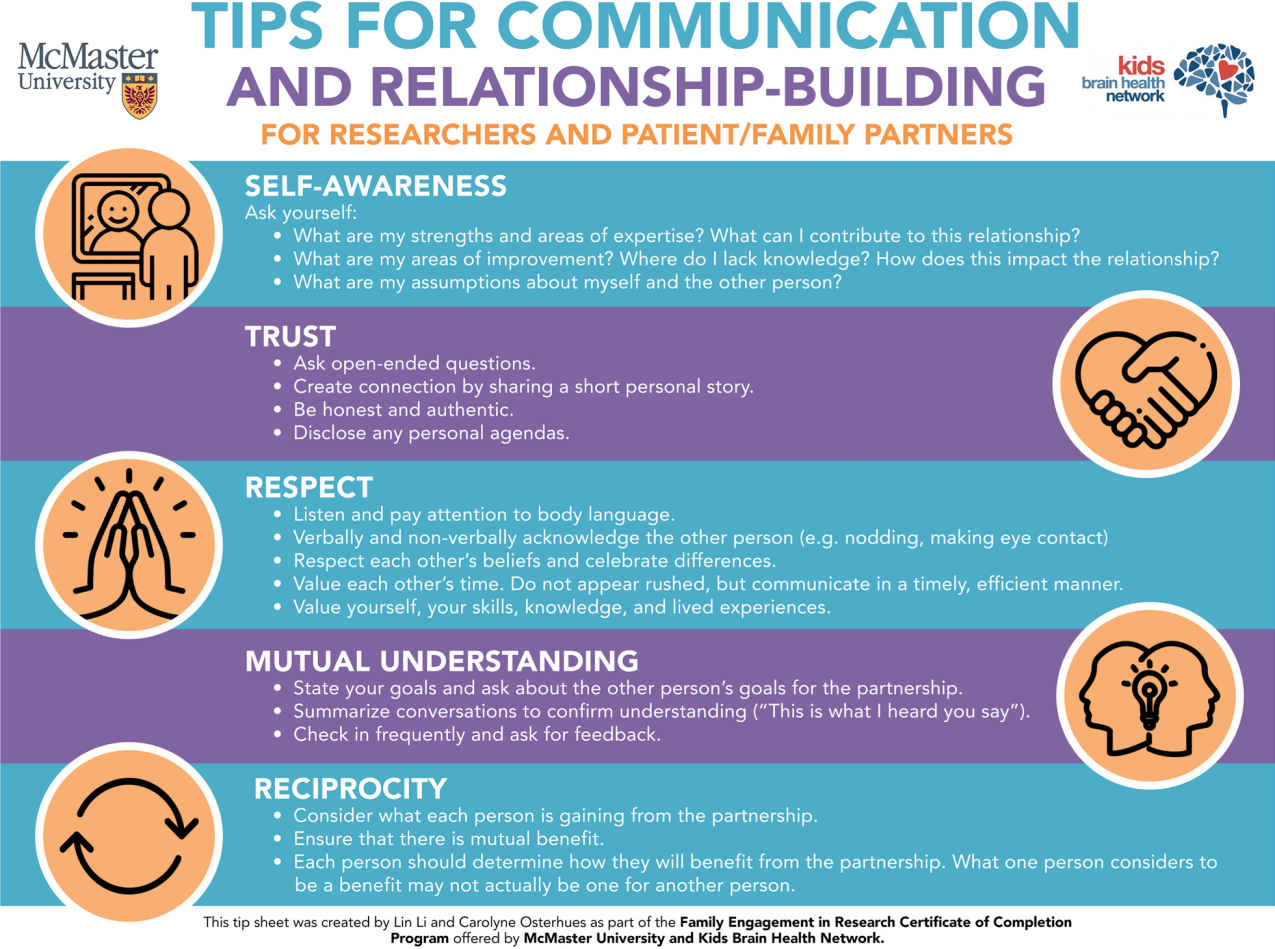
Example of an infographic developed in the FER Course as a group project

A unique component of the FER Course is that family partners and researchers are placed into small groups in Week 1 (2–4 learners) and are required to work together throughout the course to complete the weekly learning activities, and a final group project and presentation. The course is designed this way to enable co-learning, peer-to-peer mentorship, and to have an opportunity to practice working as an integrated team. The weekly group learning activities vary and include participation in the four 2-h online synchronous sessions on Zoom (Weeks 1, 4, 6, and 10) (a daytime and evening option are offered in each of the weeks) and completion of discussion board-based activities (Weeks 2, 3, 5, 7, 8, 9). The group project involves the co-creation of a knowledge translation tool or resource on FER (e.g., podcasts, videos, infographics, etc.). Common topics include communication strategies, barriers and facilitators, ethical considerations, and diversity in FER. These tools are valuable evidence- and experience-informed resources that aim to spread awareness, increase knowledge, and support the dissemination and implementation of meaningful family engagement (see Fig. 1 for an example of an infographic created by a family partner and a researcher in the FER Course) and visit the CanChild website for more examples of knowledge translation tools [24]. In Week 10, all groups co-deliver a 10-min presentation on their project and their experience of working together. Learners must complete a minimum of eight learning activities and the group project and presentation to earn the micro-credential.

#### McMaster University Certificate of Completion

When the course was first offered, all learners who successfully completed the weekly learning activities, and the group project and presentation earned a McMaster University Certificate of Completion. In September 2022, McMaster Continuing Education received permission to offer micro-credentials that build on Certificates of Completion. Micro-credentials provide recognition of learning accomplished in short, focused learning situations through which participants develop specific skills, knowledge, and competencies that address gaps and needs in particular contexts [25, 26]. In the case of the FER Course, the micro-credential that learners now receive is a way of recognizing learning related to FER and demonstrating it to others.

### Course implementation

#### Course delivery platform

From 2018 to 2023, we partnered with McMaster Continuing Education to deliver the FER Course. The course was initially built in McMaster’s online Learning Management System (Avenue to Learn) [21] and every learner accepted into the FER Course was enrolled as a McMaster Continuing Education student. In 2023, the FER team built their own learning platform to host the course. The FER team and McMaster Continuing Education have continued to work in close partnership to support the ongoing certification of the FER Course.

#### FER Course delivery team

Each cohort is co-facilitated by a teaching team of family leaders and researchers. The first two cohorts were taught by co-founders (AC, CP, DT), and as of December 2023 we have trained 19 FER Course instructors (all previous FER Course graduates) and hired a Training Director (SM) to support the growing demand for the course. Starting in the third cohort (September 2019), we hired a family leader (RM) as a Knowledge Broker. The Knowledge Broker supports and expands the FER Community through providing a welcoming, active, and supportive environment for learners before, during, and after course completion. Embedding a Knowledge Broker within the FER Course and fostering an ongoing FER Community Network is a key component of our program which is novel within POR training in Canada (see Table 6 for a summary of the key ingredients of the FER Course).

#### FER Course learners

Over the years, the size and composition of the FER Course cohorts has changed. In the early years, we started with 20 learners and have increased to up to 36 learners. Similarly, our first two cohorts were limited to family partners (e.g., parents, siblings, grandparents) of children with a NDD and research trainees (masters, PhD, and post-doctoral students). However, as awareness of the FER Course increased, we began to receive interest and accept clinician-scientists and research staff (e.g., research assistants and research coordinators), scientists (early-, mid-, and late-career investigators), and people with personal lived and living experience. We welcome individuals from diverse backgrounds and all levels of experience who have an interest in partnering in NDD and child health research.

As of December 2023, there have been 16 cohorts and 434 graduates (156 individuals with lived experience (including family partners), 234 researchers, and 44 who are both a researcher and individual with lived experience) from 20 countries (in total there have been 115 international graduates) (see Tables 2, 3, 4 and 5 for demographic information).

**Table 2:** FER Course researcher graduates

Researcher graduates	278	%
Medical student	2	0.7
Masters student	22	7.9
PhD student	64	23.0
Post doctoral fellow	39	14.0
Early-career investigator	38	13.7
Mid-career investigator	22	7.9
Late-career investigator	6	2.2
Research staff and management	63	22.7
Clinician scientist	16	5.8
Family engagement specialist/coordinator	6	2.2

**Table 3:** FER Course family graduates

Family Graduates	200	%
Parent	176	88.0
Sibling	7	3.5
People with personal lived and living experience	12	6.0
Aunt/Uncle	3	1.5
Grandparent	2	1.0

44 people are both an individual with lived and living experience and researcher, thus are counted twice. The total number of graduates as of December 2023 is 434

**Table 4:** Canadian FER Course graduate locations

Canada	319	%
Alberta	60	18.8
British Columbia	28	8.8
Manitoba	5	1.6
Newfoundland and Labrador	3	0.9
Nova Scotia	10	3.1
Ontario	193	60.5
Quebec	16	5.0
Saskatchewan	2	0.6
Other (a)	2	0.6

Other Canadian provinces with one graduate include New Brunswick and Prince Edward Island

**Table 5:** International FER Course graduate locations

International	115	%
Argentina	2	1.7
Australia	53	46.1
Brazil	6	5.2
France	4	3.5
Ireland	3	2.6
Netherlands	18	15.7
UK	4	3.5
USA	14	12.2
Other(b)	11	9.6

Other countries with one graduate include Belgium, Croatia, Finland, Hong Kong, Israel, Italy, New Zealand, Pakistan, Qatar, Spain, and Suriname

## Sustainability and Scalability

The first eight cohorts (2018–22) of the FER Course were fully funded by a 3-year project grant awarded to CanChild from KBHN. To support the sustainability of the FER Course we introduced a course fee in September 2022. We cemented our position that families are not required to pay out-of-pocket for participation in the course unless they are associated with a research project or institution that would fund their tuition. Limited needs-based funding is also available to research trainees through an application process. We offer two annual cohorts that are open for individual registration (72 spaces available).

To support the scalability of the FER Course, we developed a course sponsorship model where institutions can sponsor fully funded cohorts. Organizations that have sponsored entire cohorts to date include the Azrieli Accelerator Program at the University of Calgary; Brighter Path Project at McMaster University; and Melbourne Children’s Campus/Murdoch Children’s Research Institute and Healthy Trajectories Research Hub, Australia. The third model is a licensing agreement where an institution can independently deliver the FER Course. For example, the FER Course has been translated into Dutch (2022) and is being offered at the University Medical Center Utrecht in the Netherlands. For licensed cohorts, we offer training and consultation support for the set up and delivery of the FER Course (Table 6).

**Table 6:** Summary of the key ingredients of the FER Course

Key ingredient	Description
Co-developed and co-led	The course was co-developed and is co-led by a team of people with lived experience and researchers
Learning together (peer-to-peer mentorship)	People with lived experience and researchers learn together and mentor each other in the FER Course
Experiential learning	People with lived experience and researchers are placed in small groups and work on a group project together throughout the course
Community and ielationship-building	The course is run in small community-specific cohorts (e.g., everyone in the course has an interest in neurodevelopmental disability and child health)
Course format	An online course with a combination of asynchronous and synchronous learning spread over 10-weeks to give time for learners to build relationships and work together
Course content	Evidence-based content that is provided in various formats (e.g., peer-reviewed publications, websites, podcasts, videos, etc.) focuses on how to work together in research
Knowledge broker	A knowledge broker fosters community and networking between people with lived experience and researchers during and after the FER Course
Accessibility champion	An accessibility champion fosters an inclusive and accessible learning environment by offering accessibility consultation and support to learners, instructors, and leadership during course design and delivery

Based on demand over the past six years, the FER Course has evolved from a single 10-week course into a multi-faceted training program including a suite of learning opportunities. In 2020, CanChild received a second project grant from KBHN to support the expansion of the FER program (2020–2024). With this funding, the FER team launched two new training opportunities: Family Engagement Leadership Academy (2022) and Family Engagement Fundamentals (2023). Both courses were developed in response to training needs identified by FER Course graduates and were co-developed with graduates of the FER Course. The Family Engagement Leadership Academy is a 10-week micro-credentialed McMaster University online course that aims to provide FER Course graduates with advanced training and mentorship in how to advocate, inform, and support implementation of meaningful family engagement at an organizational and community level. Family Engagement Fundamentals is a customizable (one to five-hour workshop) that aims to spread awareness and increase foundational knowledge of family engagement. Content covered is tailored to the audience and the length of the workshop. The Family Engagement Fundamentals training serves as a springboard to additional FER learning opportunities. To date, we have offered Fundamentals training to both research and healthcare organizations. FER Course graduates from other countries (e.g., France, Spain, Brazil) have informed us that Fundamentals training will be extremely helpful in their countries, where they hope to broadly increase awareness of FER.

Additionally, while the FER Course was initially designed for family caregivers, as of September 2022, individuals with personal lived experience of a NDD or a pediatric-onset chronic health condition have taken the course. Recognizing the need for training specifically for individuals with personal lived and living experience and researchers, we began exploring the topic of youth engagement in research training in 2020 [27] and recently received a CIHR healthy youth catalyst grant (2024) to continue this work. The FER Course has also expanded to include health care providers, community leaders, and policy makers in the field of child health and NDD.

## Reflections and Conclusion

From the inception of the FER Course, our team of family leaders and researchers were committed to an equal and cooperative team partnership, which has continued throughout the course development and implementation. Over the last six years, our team has learned many key lessons through developing, delivering, and scaling the FER Course. First, the leadership support and infrastructure (e.g., funding, administrative staff time, knowledge mobilization support) from both CanChild [28] and KBHN were key facilitators to the development and sustainability of the FER Course. As the course has grown, a barrier our team has faced is keeping up with the growing demand for the course and the high cost to administer the course. To address these barriers, we have trained FER Course graduates to instruct the course, hired core staff to administer the course, and designed and implemented a business plan with a diversified revenue stream to support the sustainability and scalability of the course.

Second, while our team understood the importance of having family partners and researchers co-learn the theory of family engagement, we initially did not recognize the impact of the experiential component. The group project has been shown to be a critical component for families and researchers gaining confidence in working together in practice. Third, when we initially launched the FER Course, the instructor team consisted of family partners and researchers. Over time, we have learned the critical importance of the knowledge broker and accessibility champion roles to support the delivery of the course. Lastly, the community and relationship-building within the FER Course has been central to achieving our broader goal of creating and supporting a culture and practice of meaningful engagement within NDD and child health research. A unique aspect of our program is that we have created a growing FER Community Network where we stay connected and continue to support and collaborate with graduates well beyond the FER Course. We recognize partnership as a continuous process where we learn and grow together, not as a static, one-off event.

Since 2018, the FER Training Program has evolved from a single 10-week course into a multi-faceted training program in the field of NDD and child health research. The growing sustainability and scalability of the FER Course is a testament to how it is filling a training gap in FER, in Canada and in other countries. While our training program originated in NDD and child health, we believe our training model and materials can be successfully adapted and applied to other areas of health research.

Our vision for the FER Training Program is to transform the culture of health research by meaningfully embedding lived experience in all aspects of NDD and child health research. Many FER Course graduates become advocates, champions, and leaders in family engagement within their communities, which in turn extends the reach and impact of the FER Course. In this way, the FER Course serves as a catalyst for mobilizing a culture and practice of meaningful family engagement. To end this commentary, we share a quote from a FER Course family graduate that perfectly summarizes the essence and impact of the FER Course.

*“The knowledge and connections I gained through taking the FER Course opened up a whole new world for me. I had already been sharing our family's experiences with others through online Facebook groups, but to have the opportunity to use our lived experience to inform research was exactly what I was looking for even when I did not know it existed. Taking the FER Course with caregivers, researchers, and others showed me that we can indeed work together, provided a support network, and equipped me with skills and confidence to enter the world of family engagement in research. Today, I am an active patient and public partner on research projects and advisory groups and am actively sharing about partnering in research and connecting others to opportunities.”*

## Data Availability

No datasets were generated or analysed during the current study.

## Abbreviations

CIHR: Canadian institutes of health research
FER: Family engagement in research
KBHN: Kids brain health network
NDD: Neurodevelopmental disability
POR: Patient oriented research
